# Correction: A comprehensive scan of psychological disciplines through self-identification on Google Scholar: Relative endorsement, topical coverage, and publication patterns

**DOI:** 10.1371/journal.pone.0314923

**Published:** 2024-12-02

**Authors:** Jaap J. A. Denissen, John F. Rauthmann

The Data Availability statement for this paper is incorrect. The correct statement is: All relevant data for this study are publicly available from the OSF repository (https://osf.io/rj9ae/).
