# Supplementary material for: A comprehensive scan of psychological disciplines through self-identification on Google Scholar: Relative endorsement, topical coverage, and publication patterns
Source: PLoS One. 2024 Jan 2;19(1):e0296421. doi: 10.1371/journal.pone.0296421 (PMC10760704; doi:10.1371/journal.pone.0296421)
Supplement: S1 File — (PDF) [file pone.0296421.s001.pdf]

Supplementary Materials

Analysis of language bias in researchers' profiles

To check the biasing impact of a researchers mother's tongue, we selected “social psychology” as label and searched for profiles in languages that we are (somewhat) familiar with: Dutch, German, French, Portuguese, and Spanish. Whereas we hardly found any Dutch or German profiles using “local” labels (“sociale psychologie”, “Sozialpsychologie”), we found 40 profiles for French labels (“psychologie sociale”), 150 profiles for Portuguese labels (“psicologia_social”), and 130 for Spanish labels (“psicología_social”). It thus appears that the dominant position of Anglo-Saxon profiles in our analysis is at least partly a function of English as being the dominant academic language in a country.

Replication with expanded personality psychology keyword set

As described in the main manuscript, our analysis of personality journal editors revealed that using “personality psychology” as keyword in Google Scholar (GS) did not produce broad coverage. To achieve at least 50% coverage, a combination of three keywords would be needed: “personality”, and “individual differences” in addition to “personality psychology”. Although we did not intend maximum coverage of the total population of psychological researchers who identify with a psychological discipline, the validity of our method does require a certain degree of representativity. We therefore repeated all analyses with an expanded set of profiles.

Method

Sample

On June 17th 2021, we conducted a post-hoc search for GS profiles using the keywords “personality psychology”, “personality”, and “individual differences”. This produced 180 profiles identifying with “personality psychology”, 890 profiles identifying with “personality”, and 360

profiles with “individual differences”. Only 2 scholars identified with all three keywords, 8 identified with both “personality psychology” and “personality”; 4 with “personality psychology” and “individual differences”; and 45 with “personality” and “individual differences”. Overall, our search thus resulted in 1,375 additional unique GS profiles.

Procedure

We used the same script as used in the main analysis to crawl citations for the expanded set of GS profiles. The script that was used can be found on our OSF page: https://osf.io/rj9ae/?view_only=022e120070514a748f7a3dab07dfefb8.

Results

We first checked whether including a (much) larger set of personality profiles would change the relative frequency of multidisciplinary profiles. As can be seen in Table S1, the expanded set of personality keywords resulted in an increase in the percentage of multidisciplinary profiles in disciplines *other than* personality psychology. The percentage of personality profiles that also endorsed at least one other discipline went down, from 43% to 22%. While the new figure is still relatively high, compared to other disciplines, it no longer ranked #1 in terms of multidisciplinary focus.

As can be seen in Figure S1, the relative prominence of personality psychology also showed an interesting pattern when the expanded set of keywords was used. While the increase in absolute endorsement is of course an artefact from using an expanded set of keywords, this is much less likely the case for the shape over time: After a relative decrease from the early 80s to the mid 90s, the discipline has been on the ascendance again, with an accelerated growth rate in recent years.

Table S1
Disciplines and their numeric strength

Discipline name	Number of profiles	Percentage multidisciplinary
Psychoanalysis	190	12%
Clinical psychology	900	14%
Cognitive neuroscience	1,920	7%
Developmental psychology	670	18%
Educational psychology	420	15%

Experimental psychology	230	27%
Personality psychology	1,375	22%
Psychophysiology	350	21%
Psychometrics	610	19%
Social psychology	1,470	12%
**Merged total**	**7,637**	**8%**

Note. The numbers do not add up to the merged total number because profiles that endorsed multiple profiles were counted only once in the final tally. Also, the percentage of multidisciplinary profiles is lower than the average percentage across disciplines because of a) large differences in the size of disciplines and b) the fact that multidisciplinary profiles are counted in multiple disciplines but only appear once in the merged dataset (i.e., if all multidisciplinary profiles would endorse only two disciplines, then the average total percentage would be only half of the percentage in each discipline).

Second, we also recomputed topic endorsement across the various psychological disciplines. Note that, because of the addition of “personality” and “individual differences” as disciplinary markers, they could (by our computational logic) no longer turn up in the columns of the other disciplines. Importantly, the topics of the expanded set personality psychologists partially changed, with the topics of "leadership", "motivation", "well-being", "psychopathology", "cognition", and "health" being added, and "positive_psychology", "health_psychology", "close_relationships", "cross_cultural_psychology", and "personality_development" being dropped (the other label, “personality”, was dropped because of our computational logic; see above). With the exception of the relatively neutral swapping of “health_psychology” for “health”, the new topics were somewhat broader, and less focused on social relationships. To quantify the overall convergence in topic endorsement, we correlated the vectors representing the endorsement of labels by the original set of personality psychology, vs. the vector of endorsement of the expanded set. A Spearman rank-order correlation of $r = .49$, $p < .01$, was found between both vectors, indicating moderate topological convergence across subsets of researchers.

Table S2

Top 10 frequently endorsed topics across disciplines

Rank	All profiles	Psychoanalysis	Clinical psychology	Cognitive neuroscience	Developmental psychology	Educational psychology	Experimental psychology	Personality psychology	Psychophysiology	Psychometrics	Social psychology	Multidisciplinary profiles
#1	**emotion (3.3%)**	psychotherapy (10.3%)	psychotherapy (6.6%)	neuroimaging (6.8%)	cognitive_development (3.7%)	motivation (6.1%)	attention (6.5%)	**emotion (4.9%)**	**emotion (16.5%)**	statistics (11.1%)	political_psychology (5%)	**emotion (4.9%)**
#2	neuroimaging (2.1%)	psychiatry (6.7%)	**health_psychology (5.1%)**	memory (6.2%)	cognitive_science (3.3%)	educational_technology (5.9%)	cognitive_science (6.5%)	**assessment (4.7%)**	stress (5.2%)	item_response_theory (5.9%)	social_cognition (4.7%)	**health_psychology (3.4%)**
#3	**health_psychology (2.1%)**	philosophy (4.1%)	depression (3.4%)	attention (5.8%)	social_cognition (3.3%)	education (3.8%)	visual_perception (4.3%)	intelligence (4.2%)	emotion_regulation (4.1%)	**assessment (5.6%)**	**health_psychology (4.4%)**	**assessment (2.7%)**
#4	memory (2%)	continental_philosophy (3.1%)	mental_health (3%)	fmri (4.5%)	evolutionary_psychology (2.8%)	learning_sciences (3.8%)	eye_movements (3.9%)	leadership (4.2%)	anxiety (3.9%)	measurement (4.5%)	intergroup_relations (4.3%)	intelligence (2.7%)
#5	attention (1.9%)	trauma (3.1%)	anxiety (2.8%)	aging (3.3%)	education (2.5%)	teacher_education (3.3%)	cognition (3.5%)	motivation (3.5%)	**psychopathology (3.9%)**	methodology (3.7%)	**emotion (4.1%)**	evolutionary_psychology (2.5%)
#6	judgment_and_decision_making (1.8%)	critical_theory (2.6%)	addiction (2.6%)	computational_neuroscience (3.1%)	adolescence (2.2%)	learning_and_instruction (2.6%)	**emotion (3.5%)**	well-being (3.3%)	eeg (3.6%)	structural_equation_modeling (3.6%)	prejudice (3.1%)	**psychopathology (2.5%)**
#7	social_cognition (1.8%)	**emotion (2.6%)**	psychiatry (2.6%)	judgment_and_decision_making (3.1%)	comparative_psychology (2.2%)	self_regulated_learning (2.4%)	perception (3.5%)	**psychopathology (3.3%)**	depression (3.3%)	intelligence (3.4%)	cultural_psychology (3%)	social_cognition (2.2%)
#8	motivation (1.7%)	marxism (2.6%)	**psychopathology (2.6%)**	perception (3%)	parenting (2.1%)	learning (1.9%)	psychophysics (3.5%)	evolutionary_psychology (2.3%)	**health_psychology (3%)**	biostatistics (2.6%)	judgment_and_decision_making (2.9%)	psychotherapy (2%)
#9	**assessment (1.5%)**	**psychopathology (2.6%)**	eating_disorders (2.4%)	eeg (2.8%)	child_development (1.9%)	metacognition (1.9%)	memory (3%)	cognition (2.1%)	neuroimaging (2.8%)	quantitative_methods (2.6%)	evolutionary_psychology (2.7%)	motivation (1.9%)
#10	aging (1.3%)	cultural_studies (2.1%)	cognitive_behavior_therapy (2.3%)	language (2.6%)	autism (1.8%)	**assessment (1.7%)**	psycholinguistics (3%)	health (2%)	addiction (2.5%)	quantitative_psychology (2.6%)	gender (2.6%)	social_neuroscience (1.9%)

Note. For each discipline (columns), topics are arranged in descending order of nomination frequency.
Bold topics (**emotion**, **assessment**, **health psychology***, and* **psychopathology**) appear across multiple disciplines.

Because the topological vector is also the foundation of the factor analysis reported in the MS, we repeated this analysis with the new input. As can be seen in Table S3, this did not result in substantial changes in factor loadings.

Table S3

Factor loadings across disciplines

Discipline	"Correlational"	"Experimental"
Personality psychology	**.66**	-.03
Clinical psychology	**.59**	.03
Psychophysiology	**.52**	.28
Psychometrics	**.48**	-.08
Developmental psychology	**.47**	.10
Social psychology	**.47**	-.26
Psychoanalysis	.34	-.10
Educational psychology	.31	-.07
Cognitive neuroscience	-.01	**1.00**
Experimental psychology	.18	**.47**

Note. Factor loadings from principal axis factoring with oblimin rotation are sorted on both factors in descending order. Loadings > .40 are displayed in bold.

We also re-ran our analysis of publication productivity across disciplines, including the expanded personality discipline. Note that this can slightly revise the figures for all disciplines if the distribution of multidisciplinary profiles changes (see Table S1). The greatest change should be visible for the personality profiles, however. As can be seen in Table S4, this was indeed the case. Personality psychology's average productivity dropped a full point, from 6.55 to 5.55 publications per year, thus becoming more comparable to the other disciplines.

Table S4

Differences in publication productivity per year between disciplines

Discipline	*M*	Median	*SD*	Min	Max
Psychoanalysis	5.66	3.54	7.15	0.05	40
Clinical psychology	5.93	4.44	5.43	0.10	40
Cognitive neuroscience	4.97	3.68	4.33	0.25	40
Developmental psychology	4.35	3.64	3.24	0.08	33
Educational psychology	5.91	4.28	5.79	0.11	40
Experimental psychology	5.12	4.14	4.28	0.87	40
Multidisciplinary psychology	6.12	4.67	5.31	0.33	40

Personality psychology	5.55	4.11	5.06	0.24	40
Psychophysiology	5.84	4.37	5.13	0.50	40
Psychometrics	6.58	5.07	5.04	0.21	40
Social psychology	4.67	3.57	4.17	0.18	40

Note. Productivity describes papers published per year (appearing in Google Scholar profiles). Maximal productivity (Max) was capped at 40, which was necessary for all disciplines except for developmental psychology.

Finally, we re-ran the impact analysis for all disciplines including the expanded personality discipline. As can be seen in Table S5 and Figure S3 and as expected, most results did not change, but the coefficients for personality psychology were attenuated. Although personality psychology was still ranked highly in terms of citation progression per career year, it was on par with the citation progress of cognitive neuroscience and multidisciplinary psychology. As can be seen in Figure S3, the confidence interval for personality psychology of the line describing the association between career year and citation count became more comparable (smaller) to the confidence interval of the other disciplines.

Table S5
Comparison of impact indicators across disciplines

		Predictors				
Discipline	JCR domain	JCR Median IF	JCR Aggregate IF	Career x discipline	Career (centered) × discipline	Career × productivity × discipline
Psychoanalysis	Psychology, psychoanalysis	0.40	0.46	8.51	8.42	2.42
Clinical psychology	Psychology, clinical	1.93	2.66	22.86	23.91	3.96
Cognitive neuroscience	Neurosciences	3.05	4.02	36.79	36.91	6.05
Developmental psychology	Psychology, developmental	1.87	2.67	23.36	23.90	8.91
Educational psychology	Psychology, educational	1.42	1.91	24.49	25.58	3.67
Experimental psychology	Psychology, experimental	1.87	2.61	18.09	18.91	1.80
Multidisciplinary psychology	Psychology, multidisciplinary	1.32	2.29	39.98	41.80	5.97
Personality psychology	NA	NA	NA	38.64	39.49	5.60
Psychophysiology	Psychology, biological	2.18	2.55	21.48	22.07	3.09
Psychometrics	Psychology,	1.66	2.39	35.07	34.04	4.37

Social psychology	mathematical Psychology, social	1.62	2.08	36.60	36.06	6.96

Note. Career × discipline is the regression coefficient of a regression that predicts individual researchers' citations by their career age, their discipline, and the interaction between both variables. This results in discipline-specific beta coefficients, which are displayed in the table. For the Career (centered) × discipline coefficients, the interaction is based on (within-person) centered career age. Finally, the Career × productivity × discipline coefficient pertains to the three-way interaction between career age, discipline, and productivity.

Discussion

To critically investigate how much our findings depend on the arbitrary choice of keywords, we reverse-engineered a more inclusive set of three keywords that achieved 50% coverage of personality psychology (associate) editors. Overall, results were moderately but not perfectly robust across sets of keywords. Specifically, personality still emerged as a productive, multidisciplinary, and impactful discipline, although the difference with other disciplines was attenuated. Also, when using an expanded keyword set, the prominence of personality psychology first decreased until the mid-nineties, after which it has been steadily increasing again.

There are two possible conclusions from the somewhat attenuated pattern of findings. First, it might be that the original findings relied partly on outliers, and that the increase in sample size alleviated this issue. Evidence for this was apparent in the reduction in of confidence intervals in the revised Figure S3 as compared to the original Figure 3. However, an alternative explanation is that the expanded set of profiles partly reflect scholars that do not identity with the study of entire individuals that has been hypothesized to result in increased impact. Rather, it might be that some profiles instead included researchers who are primarily interested in individual differences in focal variables without necessarily being interested in the bigger picture. If that is true, perhaps this can explain the reduced impact for the expanded set of personality researchers.

Zooming out, we consider it very promising that a benchmark analysis (in our case: an analysis of all dedicated personality journals) produced a keyword coverage of no less than 50%. This suggests that even higher and more precise coverage is possible if more expansive machine learning algorithms are used, perhaps not only focusing on inclusion terms but also exclusion terms. For the moment, this required knowledge of personality psychology, which we have as researchers within that discipline. However, with the correct benchmarks, this work could in theory be done by computers, which might also be better able the corresponding increase in profile volumes and resulting citation crawling.

As an important caveat, we submit that there was a time between processing the expanded set of personality researchers on the one hand, and the original profiles associated with the other disciplines on the other hand. As there might be important shifts in citation volume and/or publication patterns over time, results could be biased. We urge replication of our results with methods that are more comparable across disciplines in terms of what keyword strategy is used, and the timepoint at which citations are crawled.

Impact across disciplines excluding Heckman

Table S6. *Comparison of impact indicators across disciplines, excluding James Heckman*

				Career x discipline		Career (centered) × discipline		Career × productivity × discipline	
Discipline	JCR domain	JCR Median IF	JCR Aggregate IF	B	CI	B	CI	B	CI
Psychoanalysis	Psychology, psychoanalysis	0.40	0.46	8.43	[6.8, 10.06]	8.34	[6.67, 10.01]	2.34	[2.15, 2.54]
Clinical psychology	Psychology, clinical	1.93	2.66	23.52	[21.58, 25.47]	24.75	[22.74, 26.76]	3.87	[3.63, 4.11]
Cognitive neuroscience	Neurosciences	3.05	4.02	36.8	[34.98, **38.61**]	36.92	[35.06, **38.79**]	6.03	[5.8, 6.26]
Developmental psychology	Psychology, developmental	1.87	2.67	25.26	[23.27, 27.26]	25.75	[23.7, 27.8]	9.44	[9.13, **9.75**]
Educational psychology	Psychology, educational	1.42	1.91	24.49	[22.2, 26.79]	25.58	[23.2, 27.97]	3.74	[3.44, 4.04]

Experimental psychology	Psychology, experimental	1.87	2.61	18.16	[15.58, 20.74]	18.99	[16.19, 21.79]	1.7	[1.37, 2.03]
Multidisciplinary psychology	Psychology, multidisciplinary	1.32	2.29	37.85	[35.07, **40.63**]	36.95	[34.08, **39.83**]	5.08	[4.76, 5.4]
Personality psychology	NA	NA	NA	40.82	[37.16, 44.48]	42.06	[38.33, 45.79]	10.17	[9.43, 10.91]
Psychophysiology	Psychology, biological	2.18	2.55	21.34	[18.88, 23.8]	21.92	[19.34, 24.5]	3.13	[2.82, 3.45]
Psychometrics	Psychology, mathematical	1.66	2.39	35.76	[33.68, **37.85**]	34.85	[32.68, 37.03]	5.3	[5.05, 5.55]
Social psychology	Psychology, social	1.62	2.08	35.16	[33.38, 36.95]	34.69	[32.86, 36.52]	6.27	[6.04, 6.5]

Note. Career × discipline is the regression coefficient of a regression that predicts individual researchers' citations by their career age, their discipline, and the interaction between both variables. This results in discipline-specific beta coefficients, which are displayed in the table. For the Career (centered) x discipline coefficients, the interaction is based on (within-person) centered career age. Finally, the Career × productivity × discipline coefficient pertains to the three-way interaction between career age, discipline, and productivity. Bold values represent values that fall within the confidence interval of the corresponding estimate for personality psychology.

Figure S1. Distribution of endorsements of psychological disciplines across historical time

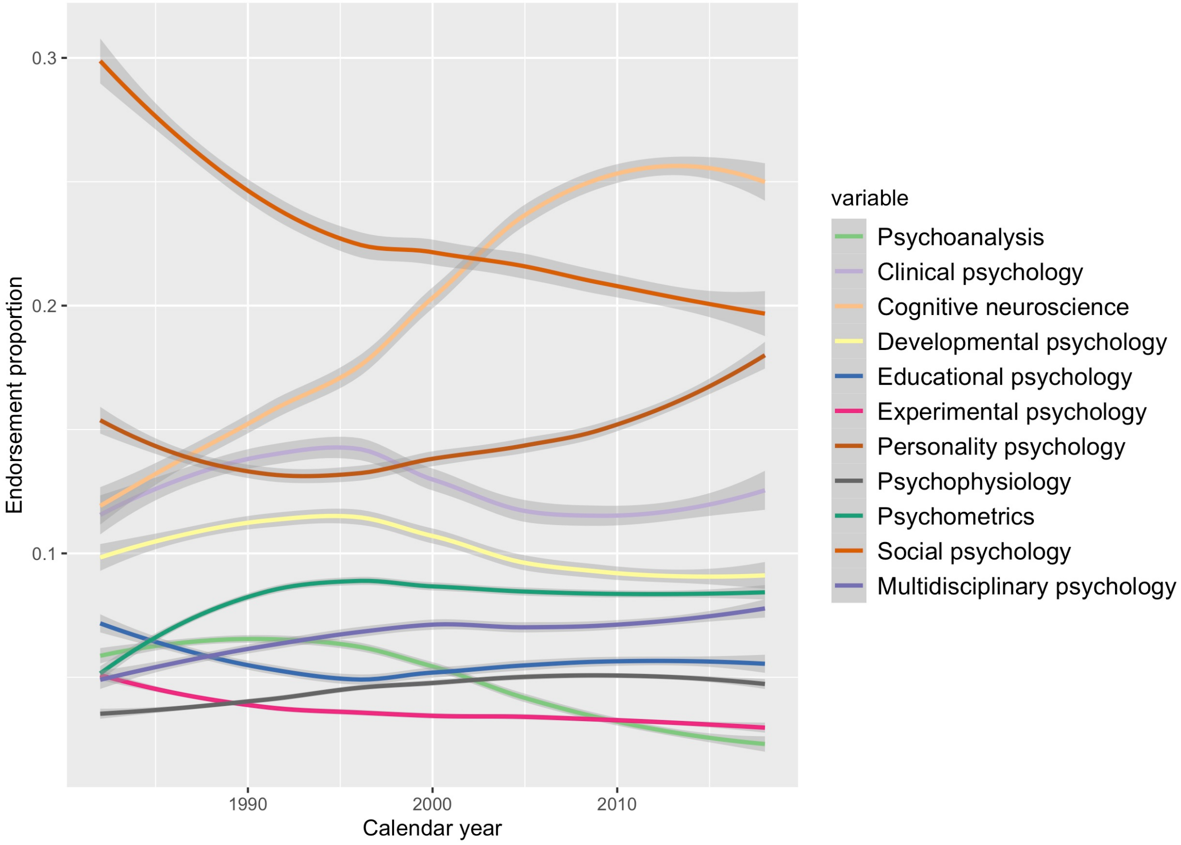

Note. Endorsement proportion (y-axis) is the fraction of all profiles that endorse a certain discipline.

Figure S2. Distribution of productivity (output per year) across disciplines

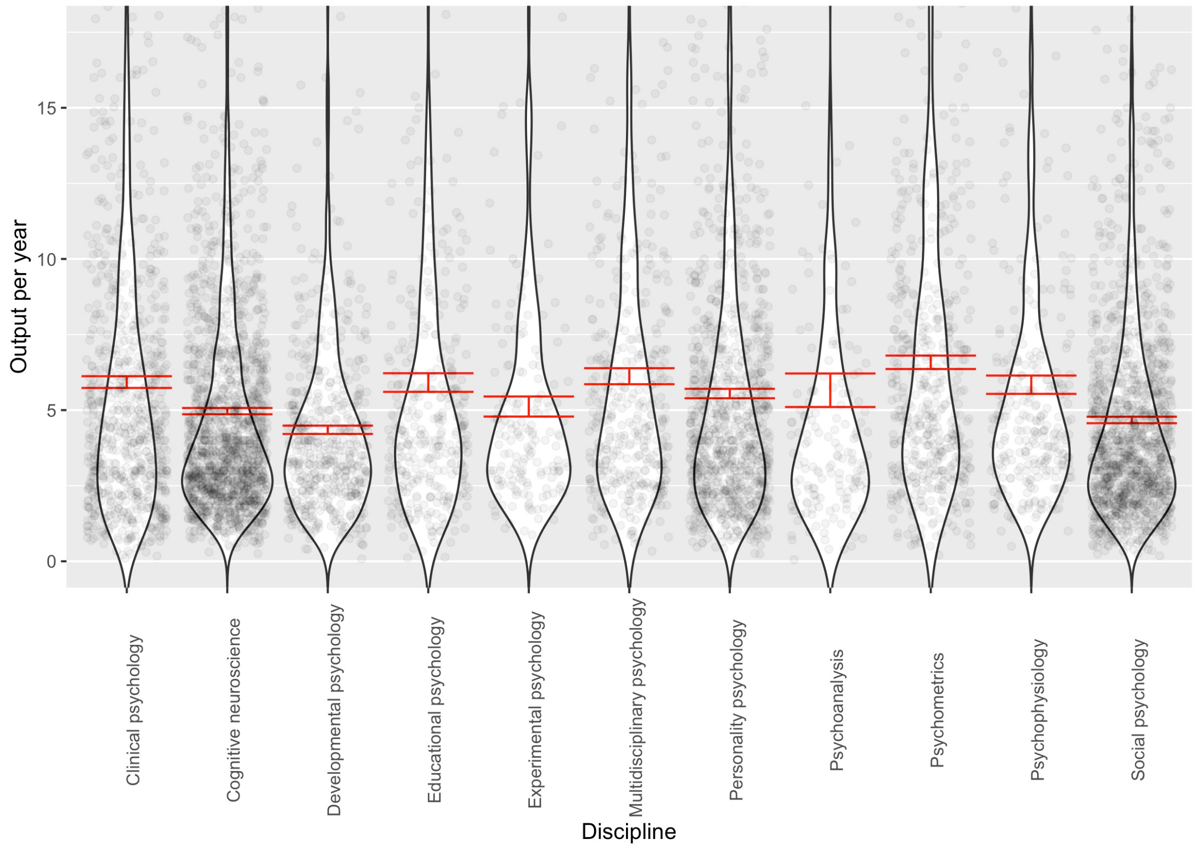

Figure S3. Development of citations across disciplines

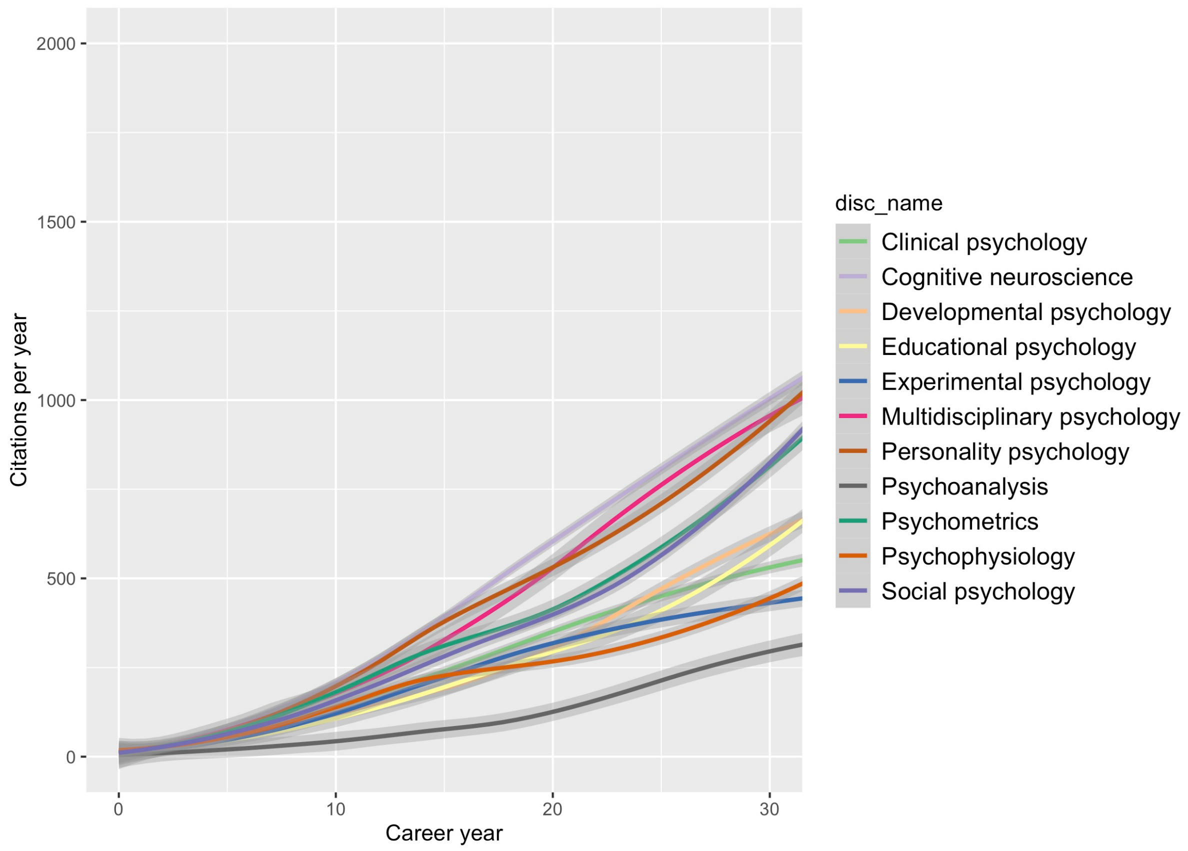

Figure S4. Proportion Ango-Saxon researcher profiles across historical time (calendar years).

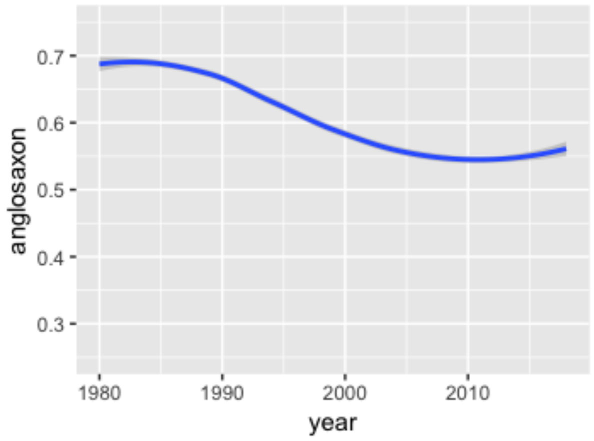

Appendix A.

Analysis of Google Scholar keywords used by (associate) editors of Journal of Personality (January 22, 2020)

Name	Google Scholar profile?	Used keywords	Used "personality psychology"?
Howard Tennen	No		No
Jonathan M. Adler	Yes	Personality	No
Colin DeYoung	Yes	Personality psychology; personality	Yes
Catherine Emily Durbin	No		No
Robin Edelstein	Yes	Personality psychology	Yes
Christian Jordan	Yes	Narcissism; self-esteem	No
Kenneth Locke	Yes	"Personality and social psychology"	No
Shanhong Luo	No		No
Donald R. Lynam	Yes	Personality; personality assessment	No
Aidan Wright	Yes	Personality; personality disorders	No
Virgil Zeigler-Hill	Yes	Social-personality	No
