# Supplementary material for: A comprehensive scan of psychological disciplines through self-identification on Google Scholar: Relative endorsement, topical coverage, and publication patterns
Source: PLoS One. 2024 Jan 2;19(1):e0296421. doi: 10.1371/journal.pone.0296421 (PMC10760704; doi:10.1371/journal.pone.0296421)
Supplement: Attachment — Submitted filename: Psychological Disciplines Scan PONE rebuttal letter.docx [file pone.0296421.s002.docx]

Dear Dr. I-Ching Lee,

Many thanks for allowing us the opportunity to revise MS PONE-D-20-40746, entitled, “A Comprehensive Scan of Psychological Disciplines Through Self-Identification on Google Scholar: Relative Endorsement, Topical Coverage, and Publication Patterns”.

We are very thankful for the many constructive suggestions by yourself and the reviewers. We have been as responsive as possible in addressing every suggestion. In the following, we outline for each of your suggestions how we have taken them into account.

We feel that addressing your suggestions has resulted in a much improved paper and look forward to your feedback on our revision!

Kind regards,

Jaap Denissen & John Rauthmann

**Editor**

1) Representativeness of the sample

1a) Although I see that you dealt with this carefully by selecting a journal (Personality Psychology) to see how the sample may correspond to the members of the editorial board as well as the authors, the correspondence is not that high.

*We have acknowledged the limited overlap in sampling as a limiting factor in the discussion (p. 33) and also expanded the scope of our benchmark search to include all personality journals (see below).*

1b) In addition, why selecting this particular journal is not clear to the readers. Why not comparing your sample (the proportions of the sub-disciplines) with the APA division memberships? (or any other association membership information that you could find). This seems to give a better evaluation of how bias or representative the sample is.

*We are not able to use the APA Division 8 because this represents an aggregation level that is too broad (it does not include a separate personality (sub-)division). However, we did consider using membership in personality associations with which we have connections (e.g., the German Differential and Diagnostic Psychology Section, or the European Association of Personality Psychology). We decided against this because current data regulations in Europe (the so-called GDPR) do not allow us to obtain personal data (including membership information). This left us with a final possibility of sending a survey to all members and track responses. While we would certainly be interested in obtaining such information, mailing lists are quite heavily used these days, which will likely depress participation rates.*

*Therefore, we ultimately settled upon expanding our selection of journals, so that our results become less dependent on arbitrary sampling decisions. Specifically, we decided to sample the editorial boards of the following major journals that are solely focused on personality (JID, JoPy, PAID, EJP, JPSP:PPID, JRP, PS). On the websites of these journals, we found 102 unique names. Of these individuals, 90 (88%) had a Google Scholar profile, and 81 (79% of all editors) used keywords on their profile. Of those 81 people, 28 (35%) used “personality” as a keyword, 10 (12%) used “individual differences” and 9 (11%) used “personality psychology”. As can also be seen in the word cloud below, these three keywords were the most frequently endorsed.*

*As the above analysis indicates, our approach only identified a minority of personality researchers with editorial roles at dedicated personality journals. The analysis suggests that selecting the keyword “personality” would have increased our sample size. We had considered this in previous versions of the MS, but decided against this for two reasons. First, there is a difference between “personality” as a subject matter, and “personality psychology” as a discipline. While it is clear that dedicated personality editors endorse “personality” as subject matter, the reverse is not necessarily true: Many other researchers (not represented in the editorial boards of personality journals) might endorse “personality” as keyword without identifying with the discipline. Second, if expanding the keyword selection for personality psychology, we should also do this for the other disciplines, which would have delayed the project by a very long time (we would have to generate suitable keywords for all disciplines and ran all analyses with a sample size many times larger). We think this task is better left for future research and we have included a call for this in our discussion section (p. 33).*

*We should also note that our aim was not so much to increase coverage of the total population but to draw a representative sample of that population. The validity of our approach therefore more strongly hinges on representativity and less strongly on coverage.*

1c) The bias of using personality psychology as an example and treating it as the bridge sub-discipline in the introduction is not grounded in current understanding. As an example, in your citation (Yang & Chiu, 2009), they grouped personality psychology with social psychology together. You treated it as if they treated personality psychology and social psychology as two sub-disciplines.

*It is correct that the Yang and Chiu publication does did not empirically separate personality and social psychology. However, this is only because personality psychology does not have its own flagship APA journal, so JPSP is a hybrid journal. While arguing for the hub position of JPSP, Yang and Chiu specifically used arguments that were based on personality psychology as science of the “whole person” (see p. 355), and resorted to ad-hoc arguments to extend this reasoning to social psychology. We now clarify this in a footnote on p. 7. More importantly, as we stated in the introduction, personality psychology has been very clearly identified as separate core discipline and its intertwining with social psychology can be somewhat seen as a historical “accident” within US psychology that has not been repeated in Europe or elsewhere*

1d) The information regarding the overlap between personality psychology and social psychology in the m.s. (e.g., on p. 18) also made me wondering whether you should treat personality psychology as an independent sub-discipline if it is often overlapped with other sub-disciplines.

*As noted above, we think there are strong reasons to treat personality and social psychology as separate disciplines. What we can imagine, however, is that disciplines like personality psychology, affective science, and motivation psychology might one day merge as explicit “boundary crossing disciplines” that have a different status than the larger mono-disciplines, such as social psychology and clinical psychology. However, we think the question of (partial) redundancy is better addressed empirically, which is why we conducted factor analysis. Indeed, personality and social psychology emerged as part of a “correlational” meta-discipline, together with other disciplines. The correspondence was not so high to merit combining disciplines, however – and our results also indicated other empirical differences between social psychology and personality psychology (e.g., the latter had a somewhat higher citation impact).*

2) Clarity of the analysis

I am not sure what has been entered in the factor analysis. It reads like topics as the information is given under the topic sentence related to topics. However, because topics could be studied in various sub-disciplines, I have no idea how the findings could be indicative of certain sub-disciplines grouped together. Please revise to make sure that information regarding each analysis could be easily followed by researchers who are familiar with the given analysis.

*We have provided more detail about the data input for the statistical analysis (p. 20). The relevant section now reads:*

*“We further proceeded to create a list with all 487 topics that had been endorsed at least 5 times (excluding the discipline labels), with columns indicating the absolute frequencies of endorsement within each discipline (log-transformed to reduce their skew). The co-occurrence of the vectors indicating relative topic endorsement in GS profiles across the 11 discipline columns can be expressed as a correlation matrix (487 topic frequencies x 11 disciplines). To examine whether this correlation matrix can be reduced to a smaller subset of “meta-disciplines”, we conducted a factor analysis. “*

3) The third is about the writing. I shared with reviewer 1’s feeling that the background of the topic is not being carefully laid out. We do not know how this research may have challenged or added to the current understanding of the field.

*We have added a paragraph to the introduction that outlines the contribution to the field in more detail (p. 11). In the discussion, we come back to this paragraph to discuss the implications of our results.*

**Reviewer 1**

1. The research problem could have been better located in the existing literature. The authors rush us into their research without an account of where research on disciplinary differences is currently at and why their approach will take us forward. It is as if they are personally well-aware of the background and expect the reader to be on the same page. A fuller introduction, although it would take a little more space, would allow us to better appreciate the value of their approach and what they have been able to add through their investigation.

*In response to this point, we have added an elaboration of the value of our approach:*

*Our approach has a number of features that set it apart from the literature. First, we take a broad approach focusing on all psychological disciplines but also go into depth regarding one discipline that is often left out of analyses: personality psychology. Furthermore, we used Google Scholar to compare disciplines, which has not been done before but has a number of advantages. For example, it allowed us to flesh out the topics that each discipline tackles, and also to identify topics that are covered by multiple disciplines. Our findings can thus give rise to more constructive suggestions for topics that have the most potential for interdisciplinary collaboration. Furthermore, our method allows for the identification of linkages with the individual scholar as unit of analysis, so novel links can emerge (e.g., if topics covary in scholarly interest profiles but are typically investigated in separate papers). Our method also has important limitations, however, which we cover in the discussion section. Finally, our analysis covers a broader timespan that has featured many important developments, for example the increased globalization of academic scholarship.*

2. I found the analysis summarised in Table 5 difficult to follow. It has to do with comparing citation rates across disciplines. There is an explanatory note to the table and the analysis is described on pp. 21-22. I have read these several times and am still having difficulty in understanding what I can conclude from the table. For example, this statement on p. 21 is too compressed: ‘For validation purposes, we added all discipline-specific regression estimates to the reference category and compared them with the discipline-specific aggregate impact figures from the 2019 JCR.’ It would help this reader to be taken through the regression analysis more slowly, from the outcome variable to the meaning of the three-way interaction.

*We have added additional explanation to the corresponding page (p. 21).*

3. The analysis summarised in Table 3 presents the factor analysis interpreted as consistent with the two-disciplines hypothesis. The first factor has no loading of .6 or stronger and the second factor has only two defining loadings (a third is a cross loading), and this is the ‘optimal’ result after dismissing a three-factor solution. The conclusion needs to be consistent with what I would characterise as a weak factor structure.

*We have added the qualification of the factor solution being “weak”. We believe that such a “weak” structure is to be expected because a stronger solution would invalidate the existence of separate disciplines.*

4. The discussion is largely speculative. The authors of course must be allowed some discretion to speculate but the extent to which they engage in this makes for a loose discussion.

4a) For example, on pp. 24-25, there is a discussion of multidisciplinarity that invokes the notion of personality psychology as a hub science and offers possible reasons for an increase in the frequency of researchers engaging with multiple disciplines. Identifying a hub science on the basis that a group of researchers is less specialised is speculative rather than persuasive.

*We agree that we might have phrased this conclusion a bit too firmly. The conclusion by Yang and Chiu was based on citation networks and empirically demonstrated that personality/social psychology represent “hubs” in psychological citation networks. What we have added to the picture is that a) personality as a topic is endorsed by multiple disciplines, b) that personality psychologists often endorse additional disciplines, and c) they are cited more frequently. We continue to believe the overall pattern of evidence is consistent with personality being a hub science, but indeed our study only highlights part of this pattern. Moreover, cognitive neuroscience also demonstrated strong citations patterns but is less multi-disciplinary, whereas experimental psychology demonstrated weak citation patterns yet researchers in this discipline often endorsed other disciplines. We have added an acknowledgement on p. 30-31.*

4b) Also on p. 25, the authors suggest that neuroimaging is a defining topic for cognitive neuroscience and psychotherapy a defining topic for clinical psychology. This is without substantiation and is not consistent with my understanding of those topics or disciplines.

*We agree that this characterization was too strong and we have replaced it with: “these topics appear central to the disciplines in question.”*

On p. 29, fashion is invoked as an explanation for the apparent decline in experimental psychology and psychophysiology, with cognitive neuroscience now the more modish label. Does this mean that experimental psychologists and psychophysiologists have not changed what they do but simply the label for self-referencing, and if so what does this mean for the validity of the conclusions that are being drawn about change?

*We were indeed not sufficiently precise in this section. In hindsight, we think that our characterization was not valid because the relative prominence of these disciplines did not decline and it is thus not possible to claim that label endorsement shifted from these disciplines to cognitive neuroscience. Instead, we now speculate that differences in access to methods might explain the difference (p. 30).*

5. My major concern is whether the methodology the authors have used is up to the weight of inference placed on it. The authors, to their credit, acknowledge limitations of their methods, both in a preliminary statement to the Results section and in the closing stages of their Discussion. These are detailed and systematic but leave the reader with real concerns about the whole approach.

5a) For example, their analysis of coverage summarised in Appendix A to me says that more than a quarter of the cases in their chosen test bed, the editors of the Journal of Personality, did not have a GS profile and only a quarter of those who did self-identified with ‘personality psychology’, which leaves me with considerable doubt about the method.

*We have now included additional personality journals that boost our conclusions, but this has not changed our overall conclusion that a “correct” disciplinary self-identification on GS is by no means a universal phenomenon. We think that this cannot be expected: For various reasons, people do not have a GS profile, fail to fill out keywords, or use different (more or less specific) keywords. More systematic research of these possible biases is needed to evaluate the merits of our novel approach. However, as we now mention on p. 33, we still think our approach has value as a “proof of principle” and express the hope that our publication will invite closer scrutiny that will address some of the doubts that the reviewer expresses.*

5b) I had less concern with the classification of disciplines used by the authors, although the addition of personality to the Web of Science system did allow this discipline to rise to ‘hub’ status.

*In a similar vein as expressed in 5a), we now explicitly encourage that future researchers will include additional disciplines, so that the generalizability of our particular classification will be empirically testable (p. 33).*

5c) It is unfortunate that the authors cannot estimate in some way the bias that might have been introduced by the voluntary nature of entries in the GS data base. With so many ‘degrees of freedom’ on the researcher’s part, comparisons seem difficult to make.

*Although it falls outside of the scope of our paper, we think that it is actually possible to quantify this bias: By systematically comparing editorial board membership of discipline-specific journals, as we did for personality psychology. We now call for such a comparison in the discussion section (p. 33).*

6. Some minor points:

p. 4. A reference to the German Psychological Society’s position would be helpful.

P. 10, bottom of page: Has ‘Question 3’ been omitted following ‘Research’?

p. 20, l. 6. ‘greater than 1’ or .1?

*We have addressed all the minor points mentioned by the reviewer.*

**Reviewer 2**

The authors may consider adding few comments concerning the following two questions.

1a) The first of which is about representative is to have a GS profile. For example, that those who identify themselves with neuroscience are more eager to create their own GS profiles than, for example, personality psychologists. Can the authors estimate approximately how many actively publishing researchers from different fields have created their personal profile in GS?

*While a systematic comparison of “profile likelihood” across disciplines is beyond the scope of our paper (it would require us to scan the editorial boards of all discipline-specific journals in psychology), we have achieved a more accurate and representative estimate for personality psychology. We now also call for comparative research into other psychological disciplines and have toned down our conclusions in the absence of corresponding evidence (see also our responses to Points 5a-d by Reviewer 1).*

1b) Another problem is categories. Because it is not mandatory there are many profiles, also among psychologists, who did not select any category.

*This is correct. In our investigation of a wider sample of prominent personality researchers, we have now included a category of “GS profile without keywords”. Fortunately, our results indicated that only 12% failed to mention a profile (inverse of 88%, p. 16).*

2) The next problem is the use of different labels. For example, individual_differences can be used instead personality especially in UK. Of course, there are many who used the both categories "personality" and "individual_differences" (like Lewis Goldberg), nevertheless there are many researchers who identified "individual_differences" as their research area (e.g. Frank Schmidt, Randall Engle, Thomas Bouchard, Dieter Wolke -- all with more than 30,000 citations). Perhaps the number of used categories also deserve a comment. There seems to be a difference if, for example, personality is identified as the only area or it is only one among several other research areas.

*This comment is spot-on. As we explain in the above, we analyzed the profiles of (associate) editors of all dedicated personality journals and found that additionally using “personality” and “individual differences” would indeed increase coverage. By have redone all our analyses using this expanded set of labels, resulting in interesting conclusions regarding the robustness of our methods.*
