# Supplementary material for: A comprehensive scan of psychological disciplines through self-identification on Google Scholar: Relative endorsement, topical coverage, and publication patterns
Source: PLoS One. 2024 Jan 2;19(1):e0296421. doi: 10.1371/journal.pone.0296421 (PMC10760704; doi:10.1371/journal.pone.0296421)
Supplement: Attachment — Submitted filename: Psychological Disciplines Scan PONE rebuttal letter rev2.docx [file pone.0296421.s003.docx]

Dear Dr. I-Ching Lee,

Many thanks for allowing us the opportunity to revise MS PONE-D-20-40746R1, entitled, “A Comprehensive Scan of Psychological Disciplines Through Self-Identification on Google Scholar: Relative Endorsement, Topical Coverage, and Publication Patterns”.

We are very thankful for the many constructive suggestions by yourself and the remaining reviewer. We have been as responsive as possible in addressing every suggestion. In the following, we outline for each of your suggestions how we have taken them into account.

We feel that addressing your suggestions has resulted in a much improved paper and look forward to your feedback on our revision!

Kind regards,

Jaap Denissen & John Rauthmann

**Editor**

1) In the previous decision letter, I have trouble accepting the analysis of the google scholar profiles as reflecting the field because the information is of a convenient sample. I asked about the representativeness of the sample. Without information better evaluate the sample, it is better to refrain from inferring that the findings reflect the field. Unfortunately, the authors were not able to provide information to better evaluate the representativeness of the sample and in my opinion should refrain from suggesting so.

*We agree that we have not drawn a representative sample of scientists and now stress this point more strongly in the discussion of our MS:*

“*The generalizability of our findings is [thus] limited to the degree that our GS sample is representative of the scholars of the studied fields. Within these limits, GS represents a unique possibility to sample thousands of scholars who self-identify as contributing to certain topics and fields – and links relevant information. This could barely be obtained otherwise, although we also note that future AI methods might perhaps automatically classify researchers based on keywords contained in paper abstracts.” (p. 33).*

*Of note, we did in fact investigate the representativeness because for the domain of personality psychology. As we report in our paper, we could improve our sampling and covered 53% of editors from major personality journals.*

*Thus, while our approach may not be perfect and leave open the exact boundaries of generalizability, it is one of the only and best approaches we have – so far.*

2) Thus, to use the publications listed in the google scholar profiles to infer productivity of the subfields is reaching and contradictory to previous findings. Yang and Chiu (2007) found that “personality and social psychology is located at the heart of psychological knowledge.” You found that “researchers identifying with psychometrics being the most productive and researchers identifying with personality psychology, cognitive neuroscience, and multidisciplinary psychology as the most impactful in terms of citation increases per additional output.” I do not see how the statement could be supported if you do not have a representative sample of psychologists and detailed data regarding citations in the field.

*While it is true that we do not have representative sampling (we address this point above), we think that our results do, in fact, square with previous analyses that have used other methods. Specifically, also Robins identified personality psychology and neuroscience as upcoming fields in their 1999 analysis. Furthermore, Yang and Chiu also identified the combined personality/social as central to psychology.*

*However, there is also a deciding difference with the Yang approach: They focused on single APA flagship journals of each discipline. However, researchers do not only publish in such journals, for example psychometric researchers also co-author publications with other researchers, for example because they ran the analyses or because they are proposing an application of a general method to solve a problem in a specific field. Our Google Scholar approach (while limited in some respects, see above) does not suffer from these limitations. We have now added a clarification about this feature of the Yang results (p. 7).*

3) Why? There are a lot of questions remaining in the google scholar profiles to reflect the field. As reviewer 3 mentioned, all publications (regardless of order of the authorship) are listed under the coauthors and double counting could be a problem especially if in some subfield a publication tends to have more authors than other subfields.

*It is true that we have not accounted for authorship inflation, and we acknowledge that it can account for differences in productivity. As stated above, for example, that many methodological scholars have many co-authorships in which they provided statistical advice to primary authors (in addition to having their own publications, of course). We now acknowledge this fact in the discussion on p. 29.*

*That said, we did explicitly correct for the number of produced papers in our account of scholarly impact, which indicates the added citation impact of one additional publication. We regard this as a strength of our method and have stressed this more clearly on p. 32-33.*

4) Also, google scholar listed publications under the same name without knowing whether it belongs to the same scholar. This could be a problem if scholars do not routinely update or check their profiles.

This can indeed occur but fortunately the problem does not seem to be very large. Recently, Tang et al. (2021) checked this for a random sample of 3,000 computer science profiles, and found that 90.5% of profiles did not contain a single publication that was falsely assigned. Misclassification was larger for more productive researchers who identified with multiple research topics, and for researchers from China (with naming conventions being partly to blame). Results also indicated that focused only on correctly classified profiles improves the validity of rankings such as ours. We call on Google Scholar to implement better methods of disambiguation (for an overview, see Sanyal et al., 2021) and for profile owners to regularly check their profiles for incorrectly classified papers (especially if they have ambiguous names, are very productive, and/or work in different fields).

*We acknowledge that this is a potential problem and have added an acknowledgement in which we also aim to quantify the likely extent of this problem (p. 35).*

Identifying Mis-Configured Author Profiles on Google Scholar Using Deep Learning

Tang, J., Chen, Y., She, G., Xu, Y., Sha, K., Wang, X., ... & Hui, P. (2021). Identifying mis-configured author profiles on google scholar using deep learning. *Applied Sciences*, *11*, 6912.

Sanyal, D. K., Bhowmick, P. K., & Das, P. P. (2021). A review of author name disambiguation techniques for the PubMed bibliographic database. *Journal of Information Science*, *47*(2), 227-254.

5) In Yang and Chiu’s article, they target papers, not authors, and have detailed data in terms of the direction (who cites who and by comparing this directionality that they are able to infer which subfield is the hub of the psychology knowledge). It is true that in their article, personality psychology is not separated from social psychology and in their view, they do not (see p. 355-356). They wrote “…personality psychology and social psychology are intrinsically connected. They share a holistic perspective on human behaviors, integrate insights from biological and experimental psychology, suggest general principles for intervention in concrete situations, and examine the contextualized nature of basic psychological processes.” In your reply letter, you wrote that “However, this is only because personality psychology does not have its own flagship APA journal, so JPSP is a hybrid journal.”

*We stand by the decision that personality and social psychology are separate disciplines. If they were intrinsically connected, they would be integrated everywhere but it is actually only the case in the USA, and perhaps some other countries (hence the decision of the American Psychological Association to merge the disciplines in one journal and collaborate in a joint social psychology/personality association, SPSP). In other countries and world regions, personality psychology is either separate (such as Germany, the UK, and Spain), or integrated with other disciplines, such as clinical psychology (this is the case in France) and developmental psychology (this is true for some universities in the Netherlands and Switzerland). We acknowledge explicitly that Yang and Chiu indeed regarded JPSP as a mixed journal (see Footnote 1), and that it requires additional argumentation to call personality a hub science (see below).*

6) Could it be that you are implying that if there were a personality psychology journal in Yang and Chiu’s database, personality psychology, instead of social psychology, would be at the heart of psychological knowledge? If this were true, it would be more consistent with your finding that personality psychology, cognitive neuroscience, and multidisciplinary psychology as the most impactful in terms of citation increases per additional output.”

*Indeed, we would speculate that a flagship journal dedicated to personality psychology would be more impactful. In Europe, where personality and social psychology are generally separate disciplines, it is useful to compare the European Journal of Personality (EJP) with the European Journal of Social Psychology (EJSP). As can be seen below, the impact factor of the personality journal is larger than the social psychology journal. It could be that this advantage is due to EJP also publishing review articles, whereas EJSP is limited to original research. When we search citations for non-review articles published during the past 5 years in both journals, EJSP has 6.93 citations per article, and EJP 8.74. These latter figures have a ratio of (non-review) papers from personality being 1,2612 times more cited. An even more direct comparison comes from JPSP itself. We searched the journal for the 257 articles with “personality” or “individual differences” as topic (field in WoS) vs. the 345 papers with other topics. The personality papers were cited 15.39 times per item, whereas the other (presumably social psychology) papers were cited 10.16 times per item (P:S ratio of 1.515). This corresponds to the relative advantage of personality psychology in our article. Because we think that this data point is not central to our main message, we have not included it in our revision but would be happy to add it to our supplementary materials if the editor desires so.*

*Table. Information from Journal Citation Reports, searched on April 21, 2022*

| *Year* | *EJP* | *EJSP* | *P:S ratio* |
| --- | --- | --- | --- |
| *2016* | *3,707* | *1,973* | *1,879* |
| *2017* | *3,494* | *2,048* | *1,706* |
| *2018* | *3,329* | *1,775* | *1,875* |
| *2019* | *3,910* | *2,415* | *1,619* |
| *2020* | *5,838* | *3,376* | *1,729* |

7) However, Allik (2013) analyzed several personal psychology journals and his findings were not consistent with this interpretation. First, in Allik’s finding, “personality psychologists from the US published the largest number of papers (4924, 57.9%) and had the largest number of citations (101 875, 68.3%).” Thus, we could not simply discount the US bias because personality psychologists in the US contributed more than the half of the publications in personality psychology.

*We are sorry about this misunderstanding. We did not want to claim that there is no separate personality psychology in the US. There are in fact many personality psychologists there, and recently there has also been the emergence of a separate US association for personality research (Association for Research in Personality, ARP). In fact, many of the researchers from our list of most impactful personality researchers were indeed affiliated with an US institutions (e.g., the top 5 personality researchers were all American: James Heckman, Richard E. Lucas, Richard W Robins, Brent W Roberts, and Todd Kashdan).*

8) In his article, he also compared those journals combined social psychology and personality and personality psychology journals, and found that personality and social psychology journals have much larger citations per papers than purely personality psychology journals.

*We believe this is a difficult comparison because the aggregation level of combined journals is greater than personality-only or social psychology-only journals. The more adequate comparison is the comparison between these latter two categories, and this bears out the findings reported in our paper, as described above. Moreover, please note that our initial units were GS profiles and not journals or single papers. This complicates the direct comparison between Allik’s findings and ours (they used different data and reside at different levels). Thus, even if there were a disagreement, it would not bear relevance because both sides can reflect different realities.*

9) Lastly, the percentage of the papers mentioned personality was low (23.6%) of all papers in these personality and social journals. Thus, your conclusion is not consistent with the previous findings and your data do not seem to be superior to the journal publication data.

*As reported above, the percentage in JPSP was higher during the last 5 years, with 42.7% mentioning “personality” or “individual differences” as a topic. Moreover, our analysis also highlighted that personality psychology is a smaller discipline than social psychology. Finally, Allik focused on articles published in dedicated journals, whereas we focused on all articles by researchers that identified with certain disciplines, which is a different level of analysis.*

10) As a result, please take out your current research question 3 “Publication Patterns of Psychological Disciplines” which speak to the disciplinary formation or the social organization of scientists rather than the interface between individual researchers and their disciplinary identification.

*We would like to refrain from doing so for two reasons. First, although we did not preregister this study, we did form a priori questions and hypotheses, and with these in mind we examined GS profiles and analyzed our data. Taking out a research question we deemed important to the entire picture would betray these intentions and also feel like cherrypicking. We also note that readers would likely anticipate, and even expect, this question and its findings, and the paper works better with them being integrated. Second, we are additionally convinced that we are in fact investigating the “interface between individual researchers and their disciplinary identification” because this represents the most direct interpretation of our raw data (publication data from individual researchers and their self-reported disciplinary identification). To assuage your concerns, we have reformulated several passages to be reflective of more careful and nuances thinking of the matter at hand.*

11) For your first question, “Research Question 1: Relative Endorsement and International Representation of Disciplines”, the research question should probably be rephrased to get at who join in the scholar google forum? In addition, I failed to see information provided for “international representation” or their subfields. As a convenient sample of psychologists, the data set is not able to tell us what the psychology as a field is about.

*We have added “(in Google Scholar)” to the description of this research question (p. 17). This should also assuage the concern that we are not able to generalize to psychology as a whole, but to the subpopulation of psychologists represented in Google Scholar. In the MS, we discuss the impact of this sampling bias at length. Further, neither we nor anyone else can quantify who joins the GS forum (that is an entirely different questions from the one we posed) because longitudinal data cannot be extracted from GS readily and there is also no easy way of identifying those not in GS. Given, however, that most researchers have GS profiles, we do not consider this research question central to understanding our Research Question 1.*

12) For your second question, in my previous letter, I have trouble understanding how you come to the classification of the topics to refer to the subfields. You entered topics (1, 0, dichotomy codes?) in factor analysis and what converged would be identified as a subfield? Shouldn’t cluster analysis or multidimensional scaling be more appropriate?

*At the level of the individual researcher, individuals could indeed be assigned 1 (vs. 0) if they endorsed (vs. not) a particular topic. Subsequently, we computed a fraction of researchers within each discipline who endorsed that topic (i.e., sum of all 1's divided by the number of researchers in the discipline). This resulted in a matrix in which the topics were the rows and the disciplines the columns (with the various fractions representing the relative endorsement of a topic per discipline).*

13) Regardless, researchers named the factors, the factor analysis itself does not. Perusing the topics listed for the subfields, many topics are overlapped. What I can guess if that the topics emerged to be several factors. However, how these factors turn out to be representing subfields is still not clear to me. Please elaborate and pay attention to the overlapping topics in different topic factors. The information could be useful and not merely something to be discounted as it could refer to the interface between researchers’ disciplinary identification.

*While it is common in factor analysis to discard “double loadings” when interpreting (and afterwards labeling) factors, you are right that topics that are represented by multiple dimensions are potentially important “interfaces” between different meta-domains of psychology. In the case of overlapping topics, this would produce a correlated factor structure, which is why we used an oblique rotation. We now highlight this more strongly on p. 20. Indeed, we highlight these overlapping topics (e.g., motivation, emotion) as a key opportunity for interdisciplinary engagement (p. 36).*

14) In Yang and Chiu and other’s papers on the topics, they typically found two dimensions: basic versus applied and population-specific versus population-general. How are these two dimensions correspond to your two factors?

*We appreciate these dimensions, and they indeed turned up in some of our topics (e.g., “adolescence” representing research with adolescent populations; “psychotherapy” representing an applied topic). As such, these topics had every opportunity to turn up as major organizing dimensions, yet they do not correspond well to the factors that we identified. We now comment on this in our paper (p. 28).*

15) (Again, is factor analysis the best analysis strategy? Why not cluster analysis or multidimensional scaling? After all, factor analysis does not work well with dichotomous data, see Kubinger, 2003). How are the findings correspond to each other using different analysis strategies?

*See above for our response why our analytical framework does in fact suit the data at hand (i.e., we used fractions instead of dummy variables).*

16) Lastly, even if you are trying to get information regarding the internet forum of psychologists, concerns raised by reviewer 3 are still valid and needed to be addressed.

*We have addressed the various points by Reviewer 3.*

17) In conclusion, I would ask you to focus your analyses and statements on the internet forum of psychologists (google scholars) and refrain from implying or stating to reflect the field. It is simply overstatement and the contradictions between your conclusions and pervious researchers do not favor you judging the quality of the data.

*We hope that our added elaborations and analyses have convinced you that our data do not point to radically different conclusions than previous researchers. That said, we happily comply with the suggestion to stress even more that our results primarily apply to the population of GS researchers, and it is an open question to what extent they might generalize to other (wider) populations.*

Kubinger, K. D. (2003). On artificial results due to using factor analysis for dichotomous variables. Psychology Science, 45, 106 – 110.

**Review Comments to the Author**

Reviewer #2: This is a valuable contribution, which I would like to recommend for publishing in its present form.

Reviewer #3: This study investigated the inter-relations of different psychological disciplines and their developmental trends via a relatively new academic metric (i.e., Google Scholar Profile) compared to previous studies based on Journals, Web of Science (Journal of Citation Report), or Scopus…etc. However, using GS profile might also cause several main problems needed to resolve to make sure the results are valid and robust.

1. Although the Limitation section had pointed out that the issue of publications on Google Scholar Profile might not be written by the same author account. Therefore, it would be better if the authors can check their datasets to know how the prevalence of this issue could happen. For instance, randomly choose 20 scholars in each subdomain and investigate the proportions of this phenomenon.

*We were actually already aware of this issue and our scripts included a check of faulty authorship, which ensured that publications not written by the focal author would be removed from the database:*

*id <- id[grep(tolower(author_name), tolower(id$author)),]*

*Accordingly, we think that this limitations actually applies less to the present study (when it pertains to false authorship claims), but it might still pertain to researchers with very common names, whose GS profiles might attract publications from namesakes without a GS profile. This issue was empirically investigated by a recent publication by Tang (2021), and we added a corresponding reference (p. 35).*

2. In addition, articles with more than one author on Google scholar Profile could also raise another issue for double-counting (or even recounting several times) their citations for the same sub-domain or even in the different sub-domains. For example, if two scholars wrote an article with high citations, one scholar identified as a personality psychologist, and the other identified as a cognitive psychologist. How the authors resolve these recounting problems? Furthermore, different recounting problems might cause different meanings for the results.

*Fortunately, this is not a problem for our method because citations are tallied per author by GS (at least for the analyses reported here). Importantly, however, in case an author endorsed multiple disciplines, he or she was allocated to a “multidisciplinary” category, so comparisons between different disciplines (both in terms of quantitative output and citations) are unaffected by the mentioned problems.*

3. Corresponding to the previous two comments, it would be better if the author could provide more related research articles in order to confirm that the Google Scholar profile analysis is stable and valid.

*We have added recent articles that address the reliability and validity of GS profiles (Tang et al., 2021; Gasparyan et al., 2017).*

4. Another issue is about the categorization procedures of subdomains in psychology. Although “Personality” played an essential role in the whole history of psychology, based on the results of the current study, it showed the lowest Google Scholar profiles (N = 120/6532) and the largest cross-disciplinary (43%) with other domains of psychology. Therefore, the highly overlapping issue of Personality psychology with other subdomains needs further illustrations for legitimacy.

*We have included personality psychology a priori because it is, as the reviewer correctly pointed out, a classical domain within psychology. The fact that it emerges as a smaller domain with a large overlap with other domains (particularly social psychology) is a novel finding, which converges with its placement within the broader “social psychology” dimension in Web of Science. We think that another result reinforces its separate status, however: As a discipline, it is ideally suited to be combined with other disciplines and can thus play a role to “bridge” subdiscipline boundaries within psychology.*
